# Monitoring and Modulating Inflammation-Associated Alterations in Synaptic Plasticity: Role of Brain Stimulation and the Blood–Brain Interface

**DOI:** 10.3390/biom11030359

**Published:** 2021-02-26

**Authors:** Maximilian Lenz, Amelie Eichler, Andreas Vlachos

**Affiliations:** 1 Department of Neuroanatomy, Institute of Anatomy and Cell Biology, Faculty of Medicine, University of Freiburg, 79104 Freiburg, Germany; 2 Center Brain Links Brain Tools, University of Freiburg, 79110 Freiburg, Germany; 3 Center for Basics in NeuroModulation (NeuroModulBasics), Faculty of Medicine, University of Freiburg, 79106 Freiburg, Germany

**Keywords:** synaptic plasticity, lipopolysaccharide, interleukin 10, transcranial magnetic stimulation

## Abstract

Inflammation of the central nervous system can be triggered by endogenous and exogenous stimuli such as local or systemic infection, trauma, and stroke. In addition to neurodegeneration and cell death, alterations in physiological brain functions are often associated with neuroinflammation. Robust experimental evidence has demonstrated that inflammatory cytokines affect the ability of neurons to express plasticity. It has been well-established that inflammation-associated alterations in synaptic plasticity contribute to the development of neuropsychiatric symptoms. Nevertheless, diagnostic approaches and interventional strategies to restore inflammatory deficits in synaptic plasticity are limited. Here, we review recent findings on inflammation-associated alterations in synaptic plasticity and the potential role of the blood–brain interface, i.e., the blood–brain barrier, in modulating synaptic plasticity. Based on recent findings indicating that brain stimulation promotes plasticity and modulates vascular function, we argue that clinically employed non-invasive brain stimulation techniques, such as transcranial magnetic stimulation, could be used for monitoring and modulating inflammation-induced alterations in synaptic plasticity.

## 1. Introduction

Inflammatory processes aim at resolving tissue damage by facilitating repair and recovery mechanisms [1]. However, unrestrained inflammation can cause damage, which may induce inflammation-associated pathologies [2]. Adaptive and acquired immune responses consist of complex pro- and anti-inflammatory signaling pathways that regulate inflammatory processes through distinct positive and negative feedback mechanisms [3]. Dysregulation of these processes may disturb physiological repair and recovery mechanisms, thus contributing to disease progression and infaust outcomes, which has been demonstrated in meningitis, encephalitis, and multiple sclerosis [4,5,6].

Two millennia ago, seminal work by Aulus Cornelius Celsus described signs of inflammation including heat, redness, swelling and pain, suggesting a prominent role of the vascular system in inflammation. Indeed, altered vascular function has been determined to be a common feature of inflammatory processes [7]. Meanwhile, cellular and molecular mechanisms that modulate vascular function during inflammation have been identified. In addition to circulating and local endothelial factors that control vascular tone and permeability, the regulation of cell migration across endothelial barriers has been recognized to be a key step in inflammatory processes [8,9,10].

In the 18th century Rudolph Virchow emphasized the disturbance of function (“*functio laesa*”) as an important feature of inflammation [11,12]. However, the factors that promote inflammation-related tissue dysfunction remained unknown. Notably, Virchow was among the first scientists to characterize a glial (non-neuronal) cell type in the brain similar to macrophages of the immune system [13,14,15]. An early study determined that these cells contribute to inflammation in the central nervous system, e.g., through phagocytosis [16]. This initial work paved the way for a deeper understanding of the function of microglia and the immune system in the brain [17].

**Figure 1:**
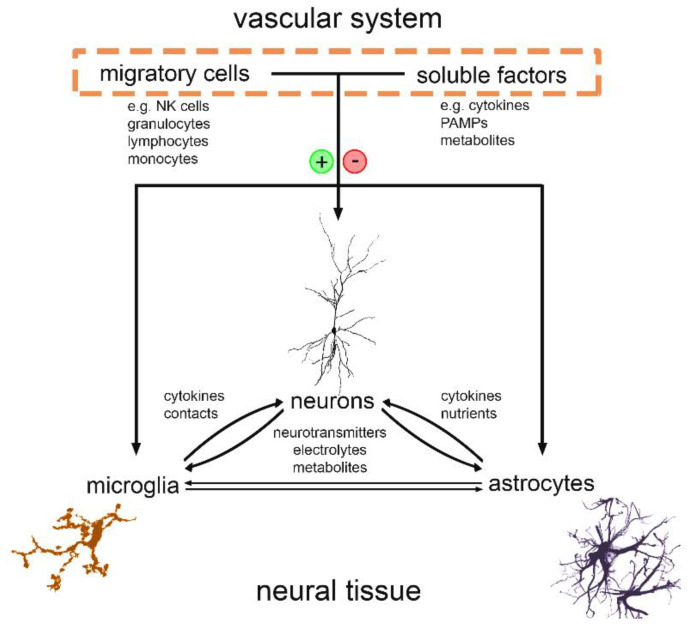
Blood–brain communication is a crucial regulatory element in neural circuit function. Neurons and glial cells bilaterally influence their function through various mediators, such as neurotransmitters, metabolites and brain derived cytokines. Moreover, soluble and cellular blood components can enter the central nervous system, when the permeability of the blood–brain interface changes. This intruiging blood–brain interaction can influence distinct features of neural circuits, e.g., network activity and the expression of synaptic plasticity.

The brain is an immune-privileged organ [18]. It is protected by the blood–brain interface (also referred to as the blood–brain barrier), which consists of tight junctions between endothelial cells, the basal lamina of the endothelial cells, and astrocyte endfeet processes [19]. A substantial body of research is currently focused on investigating the neuroimmune communication in healthy and diseased states including the role of blood–brain interfaces and more accessible meninges [20,21,22,23]. Here, we describe recent work that has identified a critical role for inflammatory cytokines in synaptic plasticity. We discuss the interplay between endogenous sources of cytokines, such as microglial tumor necrosis factor alpha (TNFα), and peripheral immune mediators (Figure 1). Finally, we highlight the potential diagnostic and therapeutic use of brain stimulation, i.e., repetitive transcranial magnetic stimulation (rTMS), which may be used as a tool for monitoring and modulating inflammation-associated disturbances of synaptic function.

## 2. Effects of Pro-Inflammatory Cytokines on Synaptic Plasticity in Health and Disease

It has been well-established that inflammation affects cognitive function and behavior, including the ability of neurons to express synaptic plasticity [24,25,26]. While the mechanisms through which pro-inflammatory cytokines affect complex brain functions have recently begun to be elucidated [27,28], little is known about their physiological relevance. Similarly, mechanisms that prevent or control inflammation, maintaining or restoring plasticity in healthy and disease states, have not yet been well characterized.

The role of the pro-inflammatory cytokine TNFα in the modulation of synaptic plasticity has been studied extensively [29,30,31,32]. TNFα is a prominent mediator of inflammation, which can be expressed by peripheral immune cells and central microglia [33,34,35]. Recent experimental work has demonstrated concentration-dependent effects of TNFα on synaptic plasticity [36]. High concentrations—as seen under conditions of inflammation–impair long-term potentiation (LTP) of excitatory neurotransmission. Conversely, low concentrations of TNFα facilitate synaptic plasticity [36]. These findings support the biological significance of TNFα-mediated synaptic plasticity in health and disease.

Robust experimental evidence has demonstrated a role for microglia in physiological brain function when inflammation is absent. Microglia assist in shaping neural connectivity by pruning synapses [37] and have been recently linked to brain homeostasis under conditions of hyperexcitability [38]. Moreover, microglia have been identified as a major source of TNFα [39]. TNFα has been shown to modulate synaptic neurotransmission by acting on astrocytes, providing evidence for a relevant crosstalk between glial cells [40]. While the precise mechanisms through which distinct TNFα levels affect microglia–astrocyte interactions and modulate synaptic plasticity have not yet been well characterized, it is conceivable that a pathological increase in brain TNFα levels—as seen under conditions of local or systemic inflammation—may induce dysregulation of synaptic transmission and plasticity.

This hypothesis has been supported by research describing the effects of the bacterial endotoxin lipopolysaccharide (LPS) on synaptic plasticity: Several studies have shown that systemic (intraperitoneal) administration of LPS promotes a substantial increase in brain TNFα levels, which impairs synaptic plasticity [34,41,42,43,44]. Consistent with these findings, we have recently shown that application of LPS directly to cultured brain tissue triggers microglial TNFα expression and leads to alterations in synaptic plasticity [34]. Further evidence comes from animal models of experimental autoimmune encephalomyelitis [45], demonstrating that IL1β, which is produced by microglia and infiltrating lymphocytes, promotes long-term potentiation but effectively prevents long-term depression of excitatory neurotransmission [46]. Accordingly, systemic and local inflammation are expected to have a major impact on brain function by triggering immune responses that induce the expression of TNFα, IL1β, and other cytokines above physiological levels.

Apparently, more work is required to determine how complex cytokine patterns affect the balance and interplay between distinct forms of synaptic plasticity in health and disease. It will be also important to gain further insights on the neuronal targets and precise cellular and molecular downstream mechanisms through which inflammation affects synaptic plasticity and brain function (c.f., [31,40,47]).

## 3. Restraining Inflammation Restores Alterations in Synaptic Plasticity

The balance between pro- and anti-inflammatory factors determines the course and the outcome of inflammatory processes in various organ systems [48,49,50]. However, in the brain our knowledge about anti-inflammatory signaling pathways and their impact on synaptic function is limited.

Among anti-inflammatory cytokines, interleukin 10 (IL10) plays a crucial role in restraining and eventually terminating inflammatory processes [51,52,53]. A deficiency or impairment in IL10 signaling has been linked with aggravated and prolonged inflammation with detrimental clinical outcomes; an example of this includes inflammatory bowel diseases [54]. Accordingly, it is well established that IL10 inhibits the production of pro-inflammatory cytokines by assembling heterodimeric receptors consisting of IL10R1 and IL10R2 at the cellular surface [55].

Recent work has linked IL10 to the modulation of excitatory neurotransmission [56,57]. Consistent with recent in vivo studies [39], our own work in organotypic brain tissue cultures showed that IL10 restores LPS-induced alterations in synaptic plasticity while limiting the expression of pro-inflammatory cytokines, including TNFα, IL6, IL1β and IFNγ [34]. It is not clear, however, whether IL10 acts directly on neurons and their synapses (c.f., Figure 1). Alternatively, IL10 may enhance synaptic plasticity indirectly by modulating microglial cytokine expression and/or microglia–astrocyte interactions.

Another major question is focused on identifying the source of IL10. Transcriptome analyses of microglia isolated from various animal disease models have failed to demonstrate IL10 expression in microglia [39]. In many studies, macrophages, natural killer-cells and neutrophils which can enter the central nervous system via the blood–brain interface have been identified as the major sources of IL10 [39,58]. Microglial IL10 expression has also been demonstrated in cultured microglia [59,60]. While these in vitro findings have been critically discussed since cultured microglia can adapt a gene expression signature that resembles macrophages [61,62], a recent study demonstrated that microglia in organotypic tissue cultures maintain their characteristic tissue imprints, such as Sall1 expression [63]. Indeed, we were recently able to demonstrate that LPS triggers the endogenous expression of IL10 in brain tissue cultures [34]. Interestingly, application of exogenous (recombinant) IL10 hampered the LPS-induced increase in IL10-mRNA in our tissue cultures [34]. These findings are consistent with a negative feedback mechanism in which circulating IL10 in the blood or peripheral IL10-expressing immune cells that enter the brain under pathological conditions suppress the endogenous expression of microglial IL10.

Together, these findings emphasize an important role for the blood–brain interface in mediating and modulating inflammation-associated alterations in synaptic plasticity. On the one hand, alterations in the blood–brain interface function, as seen for example in the context of systemic inflammation, may allow serum cytokines and other blood-derived factors to enter the brain and act either directly on neurons or indirectly by affecting neurotransmission and plasticity through activation of microglia. On the other hand, anti-inflammatory mechanisms, including migration of peripheral immune cells across the blood–brain interface, may counter and restore inflammation-associated alterations in synaptic plasticity. Whether endogenous (brain) and exogenous (blood) pro- and anti-inflammatory cytokines assert distinct effects on synaptic plasticity is currently unknown. Certainly, the stimuli driving their expression in health (e.g., modulation of synaptic plasticity) and disease (e.g., inflammation and tissue repair) appears to be distinct.

## 4. Non-Invasive Brain Stimulation as a Tool to Monitor and Potentially Modulate Cytokine Expression

Non-invasive brain stimulation techniques are widely used in research and clinical practice [64]. Among them, rTMS has been shown to induce long-lasting changes in cortical excitability by depolarizing cortical neurons through the intact skin and skull [65,66,67]. Notably, rTMS has been approved by the U.S. Food and Drug Administration (FDA) for the treatment of depression [68]. However, the cellular and molecular mechanisms through which FDA-approved rTMS protocols assert their positive effects remain unknown. Meanwhile, robust experimental evidence from animal studies (both in vivo and in vitro) has been provided that rTMS induces structural and functional synaptic changes consistent with a long-term potentiation (LTP) of excitatory neurotransmission [69,70,71]. In line with the observation that LPS-induced inflammation affects the ability of neurons to express synaptic plasticity, we recently showed that LPS also hampers the ability of neurons to express rTMS-induced synaptic plasticity [34]. Moreover, IL10 restored the ability of neurons to express rTMS-induced synaptic plasticity while limiting the LPS-induced production of pro-inflammatory cytokines [34]. These findings provide a biological basis for the use of rTMS as a diagnostic tool for monitoring inflammation-associated alterations in synaptic plasticity.

In contrast, the therapeutic effects of rTMS have been critically discussed. The rTMS-related (exogenous) induction of synaptic plasticity, i.e., LTP, which is only transiently detected in humans, might not represent a suitable explanation for therapeutic effects with a delayed onset and a duration of several weeks and months [72,73]. In this context, it is interesting to speculate that rTMS may assert its therapeutic effects by modulating microglia function. While some evidence has indicated that low intensity rTMS affects local responses of glia [74], the impact of microglia on rTMS-induced synaptic plasticity has not yet been characterized. However, microglia are known to respond to changes in network activity [75], while those changes can modulate TNFα expression at the same time [32]: Network activity and glial TNFα expression have been demonstrated to be negatively related, and TNFα promotes compensatory, i.e., homeostatic synaptic plasticity [32,76,77]. Based on this, it is possible that rTMS, which activates neurons and may also act directly on microglia, could affect complex brain function by modulating microglial cytokine expression and subsequent glial-neuron interactions.

The effects of rTMS on blood–brain interfaces is another area of open investigation. A recent study reported an rTMS-induced increase in vascular permeability, indicating increased opening of the blood–brain barrier ([78], but see also [79,80]). Although earlier studies failed to provide evidence that rTMS affects the blood–brain barrier, previous work that has shown changes in vascular permeability in the context of hyperexcitability and epileptic seizures supports the potential role of rTMS-mediated network excitation acting at the blood–brain interface [81,82]. However, it remains to be determined whether rTMS acts directly on endothelial cells, the vasculature and/or astrocytes that are part of the blood–brain interface.

Regardless of these considerations, we are confident that a better understanding of rTMS-driven effects at neuroimmune synapses [83] and blood–brain communication will be instrumental in designing translational experiments for deciphering the cellular and molecular mechanisms of synaptic plasticity in health and disease. Organotypic tissue cultures prepared from transgenic mice [84,85] and human cortical access material [86] are ideal tools to study the interplay of central and peripheral immune mediators in this context, since they are blood-free preparations that are not connected to the body’s immune system.
